# Evaluation of the Association between Paranasal Sinus Osteomas and Anatomic Variations Using Computed Tomography

**DOI:** 10.4274/tao.2020.5811

**Published:** 2021-03-26

**Authors:** Ceyhun Aksakal, Murat Beyhan, Erkan Gökçe

**Affiliations:** 1 Gaziosmanpaşa University Faculty of Medicine, Department of Otorhinolaryngology, Tokat, Turkey; 2 Gaziosmanpaşa University Faculty of Medicine, Department of Radiology, Tokat, Turkey

**Keywords:** Osteoma, paranasal sinus, variation, computed tomography

## Abstract

**Objective:**

The pathogenesis of paranasal sinus osteoma (PSO) has not been fully elucidated. It is thought that both embryological and developmental factors play a role in the etiology. The aim of the present study was to investigate the association of frequency and localization of PSOs detected on computed tomography (CT) examination with osteoma presence.

**Methods:**

In this retrospective study conducted in December 2017 through March 2020 in Gaziosmanpaşa University Faculty of Medicine, images of a total of 18,867 patients who underwent paranasal sinus, maxillofacial CT and brain CT angiography were reviewed for the presence of PSOs. Sizes of PSOs and accompanying mucosal pathologies were identified. Associations between PSOs and paranasal sinus variations were evaluated statistically compared to the control group (200 patients without PSO).

**Results:**

A total of 176 patients (0.92%) were found to have PSO. Average age of the patients with PSO was 59.9 years (range: 18–93). PSOs were unilateral in 152 patients while 24 patients had multiple osteomas. Female/male ratio was 1.1/1. PSOs were most commonly located in the frontal sinuses. Frequencies of vertical concha bullosa, secondary middle turbinate, twisted uncinate, supraorbital ethmoid cell, intersinus septal cell, ethmoidomaxillary cell, Haller’s cell, frontal sinus hypoplasia and sphenoid sinus hypoplasia were significantly higher in the patient group compared to the control group.

**Conclusion:**

Higher or lower incidence rates of some anatomic variations in the patients with PSO could be explained by the possible effects of genetic and/or environmental factor. Additional studies are needed to evaluate these possible associations.

## Introduction

Osteomas are well-limited, slowly growing osseous tissue tumors usually located in the skull, the paranasal sinuses and the mandible (1). The frequency rate of paranasal sinus osteomas (PSOs) on standard radiography and computed tomography (CT) varies from 0.42 to 3% (1, 2, 3). They are most commonly localized to the frontal and ethmoid sinuses, and quite rare in the sphenoid and maxillary sinuses (1, 4, 5). They are more common in males, with a male/female ratio ranging from 1.08:1 to 2.6:1 (5, 6, 7). About 10% of the PSOs, which develop slowly, become symptomatic and they are mostly observed as incidental events in CT (4). CT is the best examination modality to determine the presence and localization of small-sized PSOs in paranasal sinuses. In CT, they appear as dense, compact, homogeneous, round or oval-shaped and well-limited masses (4, 5, 8). Osteomas are generally solitary, and multiple osteomas are quite rare. That multiple osteomas are often seen in Gardner’s syndrome indicates the effects of genetic factors in the formation of osteomas (9).

Paranasal sinus development continues in the first three decades of life. It was argued that genetic and environmental factors could cause anatomic variations during growth and development (10). Chaiyasate et al. (11) compared monozygotic and dizygotic twins for anatomic variations and found that some variations such as concha bullosa and infraorbital cell were more common in monozygotic twins, whereas other variations such as frontal type 3 and type 4 cell were more common in dizygotic twins. These findings imply that both genetic and environmental factors could affect the anatomic variations.

The associations between PSO and clinical conditions, especially such as mucosal sinus diseases, mucoceles and nasal polyps, were investigated in earlier studies. However, studies investigating the relationship of PSO with paranasal sinus variations are quite limited. The aim of the present study was to investigate the demographic and clinical characteristics of patients whose CT examinations showed PSO, and to determine the localizations and the numbers of PSOs, as well as their associations with paranasal sinus variations.

## Methods

### Study Design and Patient Selection

**Figure 1:**
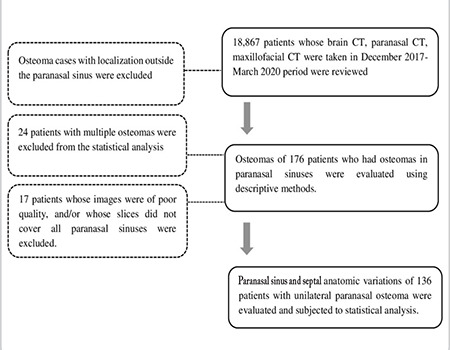
Flow diagram of patient selection CT: Computed tomography

The study was conducted in the Gaziosmanpaşa University Faculty of Medicine Training and Research Hospital with 18,868 consecutive patients older than 18 years of age who underwent paranasal sinus, brain, maxillofacial CT or brain CT angiography scan for various reasons from December 2017 through March 2020. All radiological examinations were retrospectively examined and unanimously evaluated by an otorhinolaryngologist experienced in head-neck radiological anatomy and two radiologists. Axial, coronal, and sagittal reformat images of 1.25 mm slice thickness were created from helical screening of 2.5 or 5.0-mm slice thickness in axial plane using 128-slice (Optima 660, 2016, GE) or 32-slice (Supria, 2019, Hitachi) CT machines. Images that did not include the paranasal sinuses or low-quality images with artifacts that could not be evaluated radiologically, and images of the patients with trauma, tumor or surgical history that deformed the paranasal sinuses were excluded during the comparison with the control group. An analysis of the medical records revealed that CT indications included suspect for cerebral hemorrhage and investigating the etiology of headache (brain CT), to investigate a suspected stroke (brain CT angiography), trauma and presurgical examinations for maxillofacial surgery (maxillofacial CT) and investigating paranasal sinus diseases (sinusitis, tumors, etc.) (paranasal sinus CT). The control group consisted of patients whose paranasal CT scans were performed in the period from December 2017 through March 2020 for various indications (sinusitis and paranasal tumor suspicions, etc.) and who were not found to have osteoma. The control group was determined by retrospective evaluation of these patients. While determining the control group, first, the file numbers of the 200 consecutive patients aged over 18 years who had their paranasal CT scans in the mentioned period were recorded. Then, in a second more detailed examination, paranasal variations were evaluated using the CT images of these patients. Excluded patients were illustrated by the flow diagram in Figure 1. Localization, side, size (from the axial and coronal slice), and presence of accompanying sinus diseases, polyps or mucocele were evaluated in 176 patients who were identified to have PSOs. In addition, demographic data of the patients (age and gender distribution) and the medical records of the patients were analyzed.

### Evaluation of Anatomic Variations

Anatomic variations were evaluated in patients with unilateral osteomas whose images revealed all paranasal sinus structures. Anatomic variations were also evaluated in 200 consecutive patients who underwent paranasal sinus CT examination in our hospital in the study period and had not undergone any rhinologic surgery or had nasal trauma history (control group). The anatomic variations that were evaluated are septum deviation, septum pneumatization, concha bullosa (vertical, bulbous), paradoxical middle turbinate (MT), secondary MT, pneumatized superior turbinate, pneumatized inferior turbinate, paradoxical inferior turbinate, twisted uncinate process, atelectatic uncinate process, pneumatized uncinate process, lamina papyracea dehiscence, agger nasi cell, Haller’s cell, Kuhn’s cells (type 1, 2, 3, 4), supraorbital ethmoid cell (SOEC), frontal bullar cell, intersinus septal cell (ISSC), Onodi cell, maxillary sinus hypoplasia, septated maxillary sinus, ethmoidomaxillary cell (EMC), accessory ostium, frontal sinus aplasia, frontal sinus hypoplasia, frontal sinus hyperaeration, frontal sinus pneumosinus dilatans, crista galli pneumatization, sphenoid sinus agenesis, sphenoid sinus hypoplasia, anterior clinoid process pneumatization (ACPP), pterygoid process pneumatization (PPP) and greater sphenoid wing pneumatization (GSWP).

The number and  localization of paranasal sinus variations were determined in both groups. All anatomic variations were recorded individually for both sides. Then, the associations of above-mentioned variations with osteoma in 136 patients with unilateral osteomas were evaluated statistically.

As a second analysis, the frequencies of anatomic variations in 136 patients with osteoma were statistically compared to the frequencies in the control group of 200 individuals. In this comparison, presence of the above-mentioned variations for at least once in patients with PSO and in patients of the control group were evaluated as “present.”

### Statistical Analysis

The study had a descriptive design and included the demographic characteristics, and the average and standard data of the patients. Quantitative variables were expressed as mean and standard deviation, while qualitative variables were expressed as frequency and percentage. Statistical analyses were performed using SPSS software (IBM SPSS Statistics 22, SPSS Inc., IBM Co., Armonk, NY, United States). Fisher’s Exact test was used for the analysis of qualitative data. The study was approved by the clinical research ethics committee of Gaziosmanpaşa University Faculty of Medicine (approval no: 20-KAEK-109). Informed consent was obtained from all individual participants included in the study.

## Results

### Patients

Out of a total of 337 patients with osteoma in craniofacial region, 176 patients with osteoma were identified using the flow diagram in Figure 1. Multiple osteomas were detected in 24 of 176 patients with at least one osteoma. PSO detection frequency with CT was 0.92% (176/18,867). Eighty-two of the patients with PSO were male (46.5%) and 94 were female (53.4%). Average age was 59.9 years (range: 18–93). The female/male ratio was 1.1/1. The difference between the genders for the osteoma frequency was not statistically significant based on Fisher’s Exact test (p>0.05). The measurement of the PSOs at the largest dimension varied from 2 to 50 mm. Average osteoma sizes by dimensions were as follows: anteroposterior 6.2±3.9, mediolateral 6.07±4.65 and craniocaudal 6.7±4.57 mm. As for the comorbidities seen in CT images, 21 (11.9%) patients had sinusitis findings, while nine (5.1%) patients had nasal polyp and one patient (0.5%) had mucocele in addition to osteoma (Table 1).

**Figure 2:**
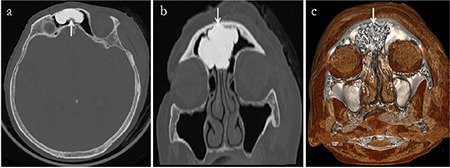
A giant osteoma (white arrows) in the right frontal sinus of an 89-year-old female patient. a) axial, b) coronal, c) volume-rendering CT images CT: Computed tomography

**Figure 3:**
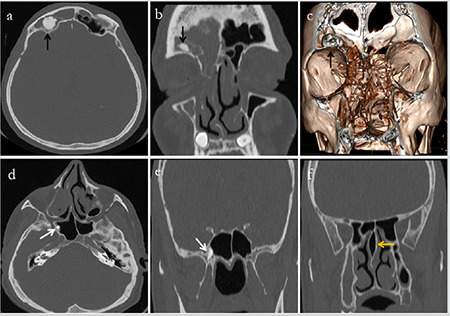
Osteoma in the right supraorbital ethmoid cell (black arrows) of a 49-year-old male patient. a, d) axial, b, e, f) coronal reformat, c) volume-rendering CT images. A second osteoma in the right sphenoid sinus (thin white arrows) and septum pneumatization (yellow arrow) CT: Computed tomography

**Figure 4:**
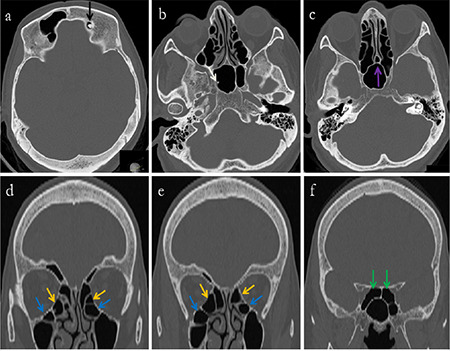
Osteoma in the left frontal sinus (black arrow), a second osteoma in the right sphenoidal sinus (white arrow) of a 64-year-old female patient. a, b, c) axial, d, e, f) coronal reformat CT images. Septum pneumatization (purple arrow), bilateral Haller’s cells (yellow arrows), bilateral ethmomaxillary cells (blue arrows) and bilateral Onodi cells (green arrows) are shown CT: Computed tomography

A further analysis of the 152 patients with unilateral osteomas revealed that the osteomas were located in the frontal sinus (Figure 2) in 74 patients (48.6%), in the anterior ethmoid sinus in 57 (37.5%), in the posterior ethmoid sinus in 15 (9.8%), in the sphenoid sinus in 3 (1.9%) and in the maxillary sinus in 3 (1.9%). PSOs were found in 65 different localizations in 24 patients with multiple PSOs (Figures 3, 4). Gardner’s syndrome was observed in one patient with multiple osteomas. Of the 217 osteoma localizations, 105 (48.3%) were on the left and 112 (51.6%) were on the right (Table 2).

The control group had a similar gender distribution (47.3% male and 52.6% female) with a mean age of 39.1±12.3 years. Statistical analysis showed significant age (p<0.05) and gender (Fisher’s Exact test, p<0.05) differences between the two groups.

### Analysis of Paranasal Sinus Variations

Table 3 summarizes the anatomic variations in patients with unilateral PSOs. In these patients, agger nasi cell and frontal sinus hypoplasia were significantly more common in the paranasal sinuses with osteoma compared to the sinuses without osteoma. No significant differences were found for other anatomic variations.

Table 4 summarizes the comparison of the anatomic variations in patients with and without PSOs. In these patients, agger nasi cell and frontal sinus hypoplasia) were more frequent in the PSO patient group compared to the control group, while paradoxical MT, pneumatized uncinate process, agger nasi cell, Kuhn’s type 2 cell and PPP were significantly more common in the control group than the PSO patient group.

## Discussion

With the widespread use of CT in the evaluation of the paranasal sinuses, the detection and treatment approaches of PSOs have been further developed (1). Although the incidence of PSO is reported between 0.01% and 0.23% in earlier studies, this frequency varies from 1 to 3% in recent studies (1, 3, 5). This is associated with the advancements in imaging methods enabling to detect very-small size osteomas (12). In the presented study, PSO frequency was found 0.92%, a value close to the lower limits reported in the more recent studies. In most earlier studies, PSO detection was done with paranasal sinus CT scanning (3, 5, 7, 12). It was argued that the incidence of PSO in sinusitis was higher (3, 7). The patients evaluated in our study did not consist solely of individuals that were admitted for sinonasal symptoms. The fact that paranasal sinus CT examination is done more commonly for sinusitis evaluation could explain the lower frequency in the presented study compared to the previous studies in the literature . This finding could also be attributed to the difference in the patient populations, hence to the fact that we studied a quite large population.

Unlike most previous studies, our study showed a female predominance (F:M=1.1:1) (1, 5, 13). Halawi et al. (12) attributed a higher incidence of PSOs detected by CT in females to the higher number of CT scans taken in women who presented with headache complaints. In our study, the mean age of the patients with PSO was 59.9 years, which was somewhat higher than the average ages in the previous studies reported in the literature (1, 5, 7). Erdogan et al. (3), having evaluated the osteoma frequencies on paranasal sinus CTs, reported a higher incidence rate in the third decade of life. The differences in the ages at which the PSOs were detected could be because our study especially included older patients whose brain CTs were taken for stroke symptoms.

Like in the other studies in the literature, the most common localization of PSOs were found to be the frontal sinuses (48.6%) also in our study (1, 5, 7, 12). PSOs were more common in the anterior ethmoid sinuses compared to the posterior ones. The pathogenesis of this frequent localization in the frontoethmoidal region is unclear. According to the embryological theory, osteomas in this region originate from the embryonal cartilaginous rest or the persistent embryonal periosteum (13). According to the traumatic theory, traumas to the frontoethmoidal region increase osteoblastic activity in this region (1). In our study, though few, some sphenoid and maxillary localizations were observed. An interesting finding in the present study was that, as in the patients with solitary osteomas, the most frequent localization was frontal and ethmoid sinus in 24 patients with multiple osteomas. However, in 24 patients with multiple osteomas, sphenoid and maxillary sinus localizations were found significantly more frequent than in the patients with solitary PSOs (Table 2). Multiple osteomas in the head can also be seen in Gardner’s syndrome, a hereditary disease (9). In the presented study, the Gardner’s syndrome was observed in one of the 24 patients with multiple PSOs.

The pathogenesis of osteomas has not been fully elucidated yet. According to the traumatic theory, traumas, especially those to the frontal region, trigger osteoblastic activity in the sinus walls. More frequent PSO incidence in men were attributed to trauma (14). Nevertheless, in the presented study, PSO frequency was higher in women. Buyuklu et al. (5) studied 243 patients with PSO and found no association between a history of trauma and the presence of osteomas. Besides, presence of osteomas in patients without a trauma history cannot be explained by traumatic pathogenesis (7). According to the infectious theory, on the other hand, chronic mucosal irritation observed in mucosal sinus diseases, such as sinusitis and nasal polyposis, increase osteoblastic activity and prepare the ground for PSO formation (7, 8). According to another hypothesis, osteoma obstructs the sinus ostium and triggers the mucosal pathology (15). Thus, it is difficult to distinguish the causality of the relationship between osteoma and mucosal pathologies such as sinusitis and nasal polyp. Halawi et al. (12) found that 30.8% of the patients with osteoma had sinusitis, 3.3% had nasal polyp and 5.3% had mucocele. Similarly, in our study most common radiopathological finding accompanying osteoma was sinusitis, while nasal polyps were less common.

Diagnosis of osteomas by X-ray radiography or CT is generally straightforward and they appear as radio-dense masses. Contrast matter use is not necessary, and they are observed in CT as well-limited, homogeneous, and generally hyperdense masses of benign nature. These characteristic radiological features differentiate osteomas from other benign tumors such as fibrous dysplasia (5). CT is the gold standard for PSO. Magnetic resonance imaging is suggested in case of intracranial or intraorbital extension (16, 17). A great majority of osteomas are incidentally detected on CT. It was reported that they could manifest symptoms when they disrupt frontal sinus drainage. Osteomas could lead to symptoms such as loss of vision, exophthalmos, diplopia, pneumocephalus and intracranial mucocele depending upon their intraorbital and intracranial compression (18). None of the patients in the present study had intracranial or intraorbital complications due to compression of osteoma. However, one patient had headache symptoms because of impaired frontal sinus drainage. Osteoma was excised using endoscopic sinus surgery in this patient.

### Evaluation of Anatomic Variations

Association of paranasal sinus anatomic variations with conditions such as nasal polyps, chronic sinusitis and antrochoanal polyp were investigated previously in many studies. Bilge et al. (19) found that frequencies of conditions such as septal deviation, concha bullosa, agger nasi cell and frontal sinus hypoplasia were significantly higher in patients with nasal polyp detected by CT compared to control group. They suggested that some variations caused obstruction in the involved region of nasal cavity, resulting in nasal polyp formation (19). Başer et al. (20) investigated the association of antrochoanal polyp on CT with anatomic variations, and found that variations such as concha bullosa, agger nasi, hyperpneumatized ethmoid bulla and Haller’s cells were significantly more frequent in the side where antrochoanal polyp was located compared to the side without polyp.

There are several studies that have addressed the effects of genetic and environmental factors on paranasal sinus anatomic variations. It was revealed that concha bullosa was more common in monozygotic twins than in dizygotic ones (11). Thus, the authors suggested that genetic factors play a role in the formation of paranasal sinus anatomic variations. In the present study, frequencies of some anatomic variations were found to be higher in patients with osteomas. For example, pneumatization of the vertical part of the MT was significantly less frequent in patients with osteoma compared to the control group (p<0.05). However, pneumatization of the bulbous part was not significantly different between the two groups. Another finding was that concha bullosa variations were not dominant on the side where osteoma was located. Janovic et al. (7) reported that concha bullosa was not associated with the presence of osteoma. Less frequent vertical concha bullosa in the osteoma group in the present study could be because of the possible compression of osteoma, which is generally observed in frontal recess region in MT. This finding implies that alongside genetic factors, neighboring pathologies could also affect anatomic variations.

It was argued that environmental factors contribute to septum deviation and to the formation of Haller’s cell and supraorbital cell (11, 21). Septum deviation has been suggested to arise from environmental causes, genetics, and trauma (22). Genetic and environmental factors also play roles in the formation of osteomas, as mentioned above. Therefore, we compared the presence of septum deviation in patients with and without osteoma, and the possible associations between the two conditions. Thus, we investigated the possibility that similar etiologies could lead to different conditions (osteoma and septum deviation) in the nasal cavity. In the presented study, no difference was found between the osteoma group and control group regarding the presence of septum deviation and the side of the osteoma localization. This finding suggests that the impact of environmental factors in septum deviation could be more prominent. Haller’s cell and EMC frequencies were higher in the osteoma group compared to the control group in our study (Table 4). Our study also showed that these two variations were not affected by the side where the osteoma was located (Table 3). Janovic et al. (7) also reported a higher incidence of Haller’s cell in patients with osteoma. Chaiyasate et al. (11) claiming that presence of Haller’s cell was more common in monozygotic twins compared to dizygotic twins, pointed to the contribution of genetic factors in the formation of this variation.

The uncinate process can have variations such as pneumatization and twisting. Pneumatization can disrupt sinus drainage and cause mucosal pathologies (23). Pneumatization frequency in the uncinate process was reported as 1%–9%, while twisted uncinate process frequency was reported as 3%–19% (24). Moreover, it was revealed that the twisted variation of the uncinate process was associated with the ethmoid sinusitis. However, there are no studies that have investigated the association of this variation with osteoma. In our study, the frequency of pneumatized uncinate processes was 11.9%, while the frequency of twisted uncinate processes was 4.4%. The frequency of twisted uncinate processes was higher, whereas the frequency of pneumatized uncinate processes was significantly lower in the osteoma patient group compared to the controls (Table 4). These variations were not associated with the side where osteoma was located (Table 3).

Kuhn (25) evaluated frontoethmoidal cells by separating them into four classes. This classification includes agger nasi cells, ISSC, the frontal bullar cell and SOEC. In the present study, agger nasi cell was significantly lower in the osteoma patient group than the control group. However, SOEC and ISSC were significantly more common in the osteoma group, while the frequency of Kuhn’s type 2 was significantly higher in the control group (Table 4). Given that, in osteoma patient group, agger nasi cell and Kuhn’s type 2 cell cases were less frequent, whereas variations closer to the frontal sinus ostium, such as SOEC and ISSC, were more common, and the frequency of frontal sinus osteomas was higher, osteomas appeared to have an association with these variations. As mentioned above, the embryological theory of PSO pathogenesis mentions that the higher incidence of PSOs in the frontal sinus and the frontal recess could be due to genetic factors. Frontal sinus localization was reported as 75.3% by Buyuklu et al. (5) and 59.3% by Larrea-Oyarbide et al. (13). Similarly, the frontal sinus (48.6%) and the anterior ethmoid sinuses (37.5%), i.e., the frontonasal region, was the most common localization of PSOs in the present study. Janovic et al. (7), on the other hand, reported the PSO incidence with frontal sinus localization as 68.3% and observed that crista galli pneumatization in the frontonasal region was significantly higher in patients with PSO. Similarly, the significantly higher incidence of variations, such as SOEC and ISSC, in the frontonasal region in the patients with PSO compared to the ones without PSO in our study, could indicate the possible effects of genetic factors. As mentioned above, this finding could be due to genetic causes, as well as environmental factors such as the compression effect of osteoma.

Frontal sinus hypoplasia and aplasia frequencies were reported to be in the range of 11.9%–40% (26). Maxillary sinus hypoplasia is less frequently observed, with a maximum reported incidence rate of 10.4% (27). Frontal and maxillary sinus hypoplasia are frequently observed in cystic fibrosis (28). To the best of our knowledge, however, there are no studies that have evaluated maxillary and frontal sinus pneumatization in patients with osteoma. In our study maxillary sinus hypoplasia was found to be higher in patients with PSO. Besides, frontal sinus hypoplasia was significantly more frequent in osteoma patients compared to the control group. Another interesting finding was that frontal sinus dysplasia was less frequent on the side where the osteoma was located (Table 3). In addition, sphenoid sinus hypoplasia was significantly more common in the osteoma patient group (Table 4). In accordance with this, PPP was less frequent in the PSO patient group compared to the control group (p<0.001). The incidence rates of ACPP and GSWP were similar in both groups. Lower frequency of sinus pneumatization in patients with PSO suggests that the patients with lower sinus pneumatization could tend to develop osteoma.

In the present study, associations between some characteristics of osteomas with anatomic variations were evaluated in a large patient group. To the best of our knowledge, there are no studies in the literature that have evaluated the associations between PSOs and such a wide range of anatomic variations as we did in the present study. Our results showed that some anatomic variations were more frequent in patients with osteomas. Especially vertical concha bullosa, secondary MT, twisted uncinate process, SOEC, ISSC, EMC, Haller’s cell, frontal sinus hypoplasia and sphenoid sinus hypoplasia were more common in patients with PSO, while paradoxical MT, pneumatized uncinate process, agger nasi cell, Kuhn’s type 2 and PPP were more common in patients without PSO.

Our study had some limitations. First, since this was a retrospective study, the clinical findings associated with osteomas could not be comprehensively analyzed. This is because as well as the patients with a paranasal sinus CT, the study also included the patients whose CTs were taken for stroke and similar intracranial reasons. It was difficult to distinguish whether their headache was associated with osteoma. Another limitation of the study was the presence of statistical difference between the study and control groups in terms of age and gender.

## Conclusion

Our results suggest that patients with osteoma presented with a higher frequency of anatomic variations of the paranasal sinuses compared to patients without osteoma. PSO was associated with concha bullosa, secondary MT and some other anatomic variations. These associations could be due to genetic as well as environmental factors. Future studies on this area could better reveal these associations.

**Main Points**

• Genetic or environmental factors are among the possible factors affecting PSO formation.

• Patients with paranasal sinus osteoma have a higher rate of paranasal sinus variations than patients without paranasal osteoma.

• The paranasal sinus osteoma was associated with vertical concha bullosa, secondary middle turbinate, twisted uncinate process, supraorbital ethmoid cell, intersinus septal cell, ethmoidomaxillary cell, Haller’s cell, frontal sinus hypoplasia and sphenoid sinus hypoplasia, all of which showed significantly higher prevalence in the osteoma group compared to the control group.

## Figures and Tables

**Table 1:**
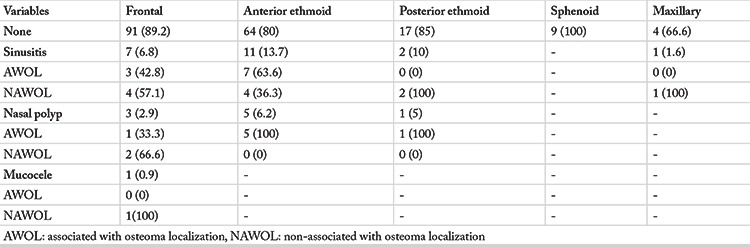
Association of osteoma localizations with sinonasal mucosal pathologies

**Table 2:**
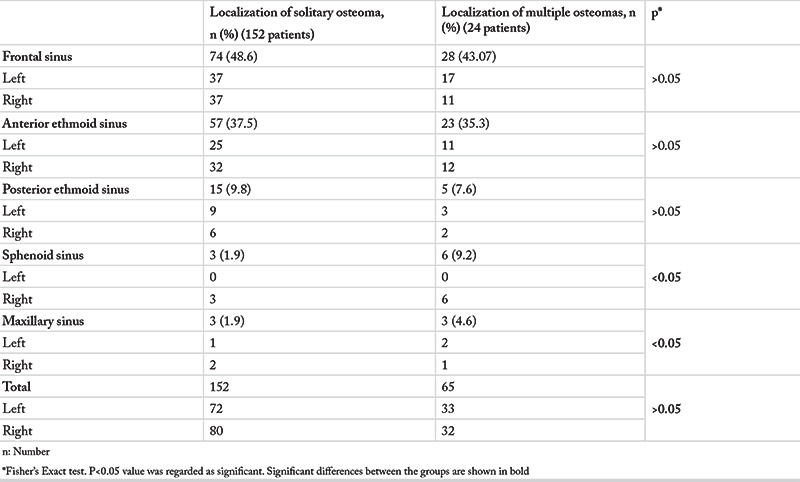
Location of the paranasal sinus osteomas

**Table 3:**
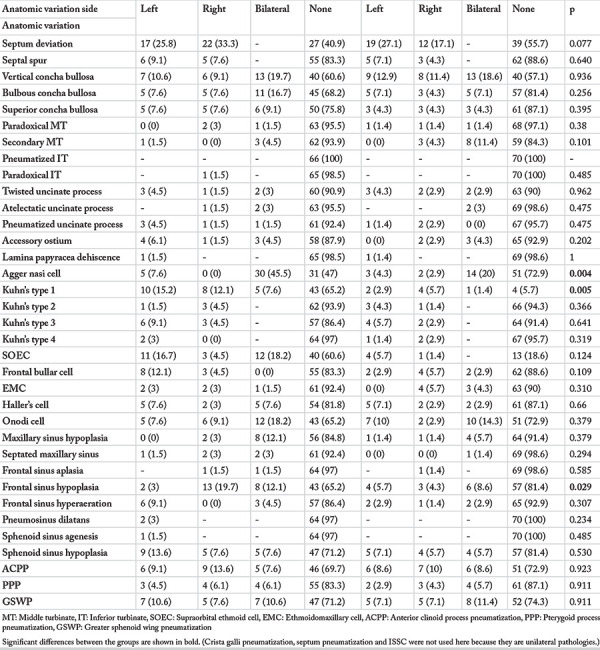
Distribution of anatomic variations based on the localization of unilateral osteomas (comparison of the anatomical variations of the side with the osteoma and the side without the osteoma)

**Table 4:**
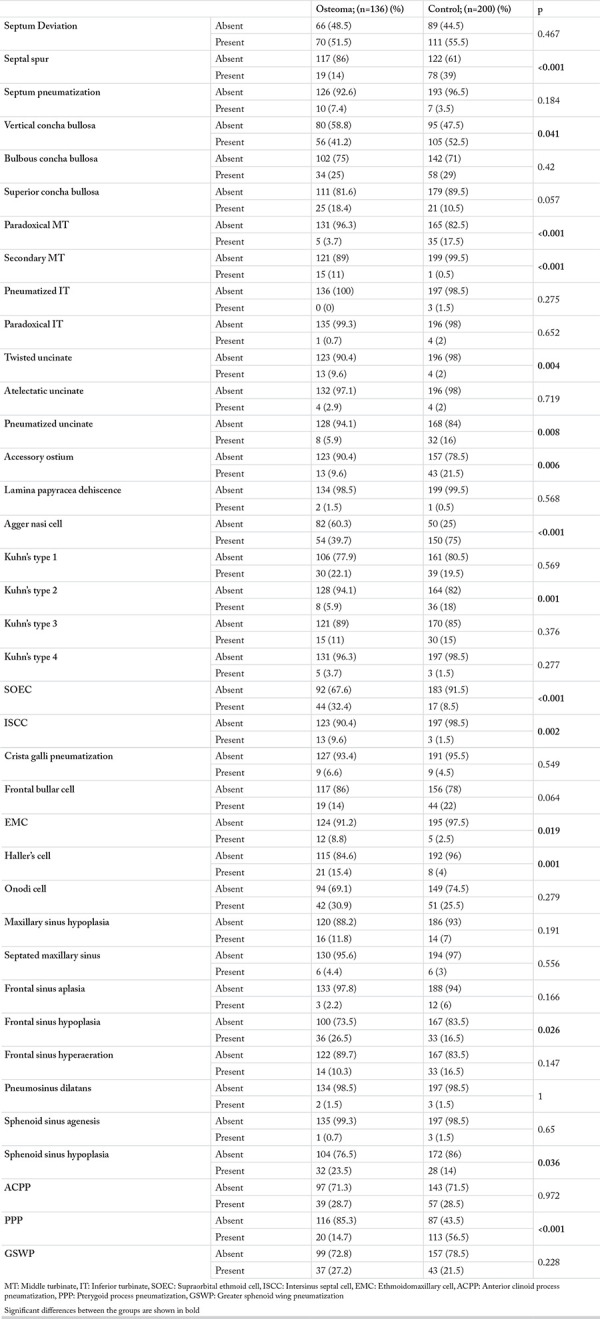
Comparison of the distribution of anatomic variations between the osteoma patient group (with a single osteoma) and the control group
